# Microservice Workflow Scheduling with a Resource Configuration Model Under Deadline and Reliability Constraints

**DOI:** 10.3390/s25041253

**Published:** 2025-02-19

**Authors:** Wenzheng Li, Xiaoping Li, Long Chen, Mingjing Wang

**Affiliations:** 1 School of Computer Science and Engineering, Southeast University, Nanjing 211189, China; xpli@seu.edu.cn (X.L.); longc@seu.edu.cn (L.C.); 2 School of Data Science and Artificial Intelligence, Wenzhou University of Technology, Wenzhou 325000, China; wangmingjing.style@gmail.com

**Keywords:** workflow scheduling, microservice workflow, container environment, cost-optimized scheduling

## Abstract

With the continuous evolution of microservice architecture and containerization technology, the challenge of efficiently and reliably scheduling large-scale cloud services has become increasingly prominent. In this paper, we present a cost-optimized scheduling approach with resource configuration for microservice workflows in container environments, taking into account deadline and reliability constraints. We introduce a graph deep learning model (DeepMCC) that automatically configures containers to meet various service quality (QoS) requirements. Additionally, we propose a reliability microservice workflow scheduling algorithm (RMWS), which incorporates heuristic leasing and deployment strategies to ensure reliability while reducing cloud resource leasing cost. Experiments on four scientific workflow datasets show that the proposed approach achieves an average cost reduction of 44.59% compared to existing reliability scheduling algorithms, with improvements of 26.63% in the worst case and 73.72% in the best case.

## 1. Introduction

The rapid advancement of information technology has popularized microservice architecture and containerization in cloud application development and deployment [1]. This trend is driven by the demand for enhancing system flexibility, scalability, and efficient resource utilization. By decoupling complex services, microservice architecture enables the independent development, deployment, and scaling of individual services, significantly improving the agility of cloud applications. However, the adoption of microservice architecture also introduces a variety of challenges, particularly in the areas of resource configuration and scheduling.

In terms of microservice resource provisioning, existing research primarily focuses on elastic resource configuration based on load predictions for individual services or specific types of services. However, microservice applications with task dependencies, often represented as workflow models using Directed Acyclic Graphs (DAGs), exhibit a higher level of complexity than independent tasks. The resource configuration for these tasks requires a comprehensive understanding of service dependencies and Quality of Service (QoS) requirements. Unfortunately, Most researchers do not take into account the resource configuration for complex workflow in scheduling, and neglect the reusability of configuration experiences, leading to inefficient resource utilization.

In terms of microservice workflow scheduling, many cloud workflow scheduling algorithms lack the resource failure model and not account for scheduling reliability. Resource failures, such as server crashes or network disruptions, can lead to diminished system performance, premature termination of program execution, and even data loss. They ultimately result in an increased number of tasks missing their deadlines, higher failure rates, and severely compromised reliability and stability in cloud computing. Due to the lower level of isolation on containers, their failure rate is higher than that on virtual machines. Therefore, it is essential to consider reliability requirements in microservice scenarios.

Moreover, containerization offers a lightweight and efficient way for deploying microservice, and they also introduce a dual-layer resource structure that consists of both virtual machines and containers. This two-tier resource structure complicates the cost optimization process, as it requires balancing trade-offs among resource utilization, performance, and cost. Traditional virtual machine allocation strategies struggle to accommodate the diverse and dynamic configurations of containers. These traditional strategies rely on limited virtual machine resources and fail to leverage the flexibility provided by containerization.

In summary, microservice architecture and containerization have significantly altered the scheduling system in terms of task scale, resource structure, and execution reliability requirements. The limitations of traditional scheduling system has prompted a shift toward more intelligent approaches [2], such as utilizing advanced AI models (e.g., deep learning and reinforcement learning). This paper presents an intelligent scheduling approach for scheduling microservice workflow on container environment, integrating graph deep learning with heuristic methods. The specific contributions of our work are as follows:

- Precise Resource Configuration: A deep learning model for microservice container configuration (DeepMCC) is designed. Because of the advantages of Graph Neural Networks (GNN) in processing graph-structured data, the approach efficiently configures container resources for each service to meet QoS requirements.
- System Reliability Enhancement: A container replication strategy is employed to enhance system redundancy and reliability. Additionally, the container migration strategy is designed to improve resource utilization.
- Cost-optimized Scheduling: For the dual-layer virtual resource environment of containers and virtual machines, a reliability microservice workflow scheduling algorithm (RMWS) is proposed. This algorithm integrates container configuration, fault tolerance, and container migration to optimize cost. Experiment demonstrate that RMWS can minimize cost while ensuring reliability compared to relevant algorithms.

This paper introduces several innovative contributions to the field of microservice workflow scheduling in containerized environments. Firstly, it presents DeepMCC, a novel deep learning model tailored for precise container resource configuration in microservice architectures. Secondly, this paper enhances system reliability through the implementation of a container replication strategy, which boosts redundancy and resilience against resource failures. Lastly, the proposed RMWS algorithm represents a significant advancement in cost-optimized scheduling for microservice workflows. The approach distinguishes itself from existing algorithms, demonstrating superior performance in managing the intricacies of modern cloud environments.

The rest of the paper is organized as follows. The related work is described in Section 2. Section 3 shows models and problem formulation. Section 4 describes the proposed methods. Experimental results are shown in Section 5. Finally, conclusions and future research are detailed in Section 6.

## 2. Related Work

### 2.1. Workflow Scheduling

Workflow scheduling is an optimization problem of mapping tasks to resources, which involves sequencing tasks and allocating resources to optimize performance metrics, including execution time, resource utilization, and cost.

In the study of workflow scheduling considering deadline constraints, researchers focus on ensuring that tasks are completed before the deadline, while the scheduling objective is to minimize cost. Wu et al. [3] introduced the concept of probability-based upward ranking and designed two algorithms, ProLiS and L-ACO, which consider both task urgency and cost factors to achieve cost optimization. Chakravarthi et al. [4] employed a novel encoding scheme and a well-designed population initialization strategy to propose a firefly-based metaheuristic algorithm called CEFA. Sahni et al. [5] considered dynamic performance changes in cloud resources and proposed a high-performance JIT-C scheduling algorithm. Toussi et al. [6] proposed the EDQWS algorithm based on the divide-and-conquer approach.

In the study of workflow scheduling considering cost and budget, Wu et al. [7] proposed a cluster-based heuristic algorithm called PCP-B2, which balances budget allocation among PCPs. Ghafouri et al. [8] proposed a heuristic algorithm called CB-DT that uses backtracking to assign critical tasks to faster resources and non-critical tasks to lower-cost resources, optimizing execution time while satisfying budget constraints. Faragardi et al. [9] extended the classic HEFT algorithm to propose the GRP-HEFT algorithm, which consists of a resource provisioning mechanism and a scheduler.

Although these studies have made significant progress in workflow scheduling, they overlook the unique challenges presented by microservice architecture, such as low scheduling efficiency caused by the scale of tasks and the resources.

### 2.2. Microservice Workflow Scheduling

In the context of microservice scheduling, Gu et al. [10] and Guerrero et al. [11] focused on container deployment issues. He et al. [12] proposed a greedy-based algorithm for rapid deployment and continuous delivery of microservice in cloud and edge computing environment by modeling it as a Quadratic Sum-of-Ratios Fractional Problem. Bao et al. [13] addressed the performance issues of independent microservice by establishing a comprehensive model and making accurate predictions. Wang et al. [14] proposed the Elastic Scheduling of Microservice (ESMS) method, which combines task scheduling and automatic scaling to meet deadline constraints while minimizing the cost of virtual machines. Li et al. [15] introduced a heuristic algorithm called GSMS to minimize execution cost while satisfying deadline and reliability constraints. Abdullah et al. [16] presented the MSDSC framework to enhance the security of edge systems. Yu et al. [17] proposed a reinforcement learning algorithm with reliability constraints (WS-CCR).

Table 1 shows the relevant work on microservice workflow scheduling over the past five years (journal papers, excluding conference papers). Most scholars tend to design heuristic methods and there is currently no research that considers deadline, cost, and reliability simultaneously. The research presented in this paper fills this gap in microservice workflow scheduling.

Apart from heuristic methods, the recent advancement of Graph Neural Network (GNN) has presented a new opportunity to tackle resource allocation challenges. GNNs possess powerful capabilities in representing graph data, are not limited by the number or order of nodes, and excel in inductive learning, making them instrumental in graph analysis problems. Their successful applications in diverse domains have demonstrated their potential in addressing graph-structured problems [18]. Wang et al. [19] employed a GNN to mine correlations and predict service usage probabilities for task solutions, and then efficiently construct initial solutions using PN-based reinforcement learning. Liu et al. [20] proposed a dynamic graph neural network based model called DySR to tackle the evolution of service and the semantic gap between services and mashups. Dong et al. [21] designed an adaptive fault-tolerant workflow scheduling framework (RLFTWS) using deep reinforcement learning, balancing makespan, resource usage, and achieving fault tolerance. Inspired by these developments, this paper explores the GNN model to map task resource requirements to container configurations.

### 2.3. Fault-Tolerant Strategies in Scheduling

To enhance fault tolerance of workflow scheduling with reliability requirement, the primary research focus is on adopting replication strategies to generate multiple duplicates of tasks to ensure their smooth execution. Passive replication involves re-executing a backup task on new resources when the primary task fails [22], but may result in deadline violations. On the other hand, active replication requires multiple copies of each task to run simultaneously on different resources [23,24,25,26] to ensure that at least one copy can successfully complete.

Early studies use a fixed number of replicas to tolerate the maximum fault rate [23,24], leading to high and unnecessary execution cost. To address this issue, subsequent research introduced the concept of quantitative active replication, which optimizes cost by dynamically adjusting the number of replicas for different tasks. Zhao et al. [25] proposed an algorithm that minimizes the number of replicas while satisfying reliability requirements, thereby reducing cost. Xie et al. [26] also considered the trade-off between the number of replicas and cost, proposing the CGM algorithm for cost optimization under reliability constraints.

Apart from replication-based fault tolerance methods, some studies have adopted resubmission strategies to ensure the reliable execution of workflow [27]. Rescheduling for fault tolerance mainly involves reallocating resources for tasks that cannot be executed smoothly due to resource failures during execution, thereby ensuring the successful completion of these tasks. This strategy is suitable for tasks with low fault rates and redundant time.

Whether utilizing replication or resubmission as fault-tolerant strategies, these studies primarily focused on cloud resource scenarios with a single-layer resource structure of virtual machines, and there is almost no research on scenarios involving the dual-layer resource structure of both containers and virtual machines.

## 3. System Architecture and Problem Description

### 3.1. System Architecture

**Figure 1:**
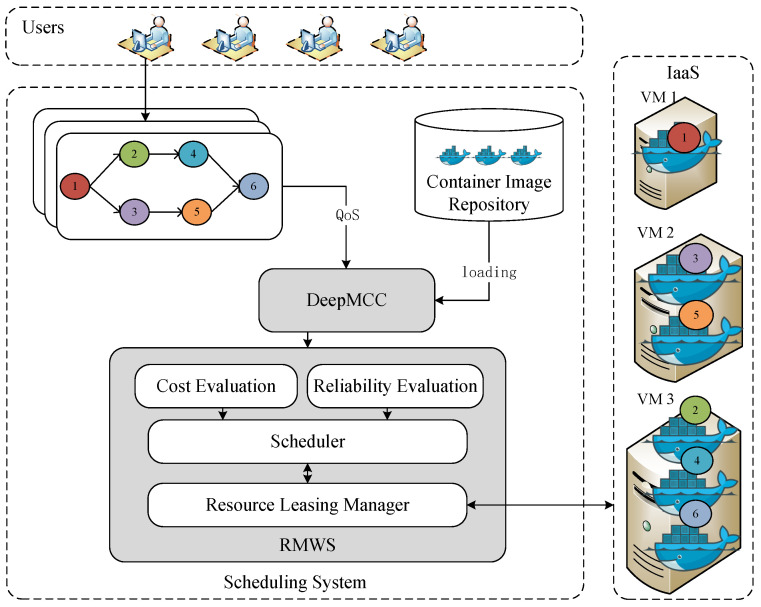
System architecture.

The system architecture is shown in Figure 1. Applications with user specified deadlines and reliability requirements are submitted to the scheduling system. Firstly, a deep learning model called DeepMCC receives workflow information, which includes the QoS (Quality of Service) requirement information. DeepMCC analyzes the resource characteristics of each workflow subtask and recommends appropriate container configurations. Subsequently, the workflow tasks with container configurations are sent to the RMWS module. The execution cost evaluator assesses the cost based on cloud service provider billing, while the reliability evaluator generates task replicas that meet reliability requirements using the replication fault-tolerant strategy.

As one of the core components of the system, the scheduler is responsible for comprehensively considering task characteristics, resource allocation, execution costs, and reliability requirements to develop the optimal scheduling strategy. The resource leasing manager is responsible for dynamically managing the leasing and release of cloud resources, while also monitoring the performance and status of resource utilization.

### 3.2. Workflow and Resource Model

A microservice workflow application is represented as a DAG (Directed Acyclic Graph) W=(V,E), where *V* denotes the set of tasks and *E* represents the set of stask dependencies. The computational workload of a task $v_{i}\in V$ is denoted as $w_{i}$. $e_{i,j}\in E$ represents an edge between tasks $v_{i}$ and $v_{j}$, meaning that $v_{j}$ can only start after the completion of $v_{i}$. The edge $e_{i,j}$ is associated with $data_{i,j}$, which indicates the size of data transferred from $v_{i}$ to $v_{j}$. $v_{i}$ is described as a predecessor task of $v_{j}$, and $v_{j}$ is described as a successor task of $v_{i}$. The set of predecessor tasks and successor tasks of $v_{i}$ are denoted as $pred(v_{i})$ and $succ(v_{i})$, respectively. $v_{entry}$ and $v_{exit}$ refer to tasks that have no predecessor tasks and no successor tasks, respectively Table 2. By adding two virtual tasks with an execution time of θ at the beginning and end of the workflow, it is assumed that the workflow has only one $v_{entry}$ and one $v_{exit}$.

The execution cost of a virtual machine is linked to its type and charged based on a unit time cost (Budget Time Unit, BTU). If an hourly-based cost model similar to Amazon EC2 is used to charge customers for renting virtual machines, any duration exceeding one hour is rounded up to the nearest hour.

The resource pool is represented as a set of virtual machines $M=\{m_{1},m_{2},\ldots \}$. The computing resource vector configured for the virtual machine instance $m_{k}$ is denoted as ${\vec{R}}^{a}\left(k\right)=\left(R_{1}^{a},\ldots ,R_{N_{r}}^{a}\right)$, where $N_{r}$ represents the total number of computing resource types (e.g., CPU, RAM, etc.). Each task $v_{i}$ is encapsulated in a container $c_{k}$, which is deployed on a virtual machine $m_{l}$ and consumes a specific amount of resources denoted as $\vec{R}\left(c_{k}\right)$.

Similar to most microservice workflow scheduling algorithms [9], it is assumed that only one microservice can execute within a container at any given time. When task $v_{i}$ is assigned to container $c_{k}$, its execution time $ET(v_{i},c_{k})$ is calculated as follows:

(1) $$\begin{array}{c} ET\left(v_{i},c_{k}\right)=\frac{w_{i}}{speed(c_{k})}, \end{array}$$

assuming all virtual machines are located in the same physical region of the cloud environment, with the bandwidth between virtual machines denoted as $bandwidth_{x,y}$ and the transmission delay as $delay_{x,y}$. The data transfer time $TT(v_{i},v_{j})$ between task $v_{i}$ and its successor task $v_{j}$ is calculated as follows:

(2) $$\begin{array}{c} TT\left(v_{i},v_{j}\right)=\left\{\begin{array}{cc} \frac{data_{i,j}}{bandwidth_{x,y}}+delay_{x,y}, & \;\mathrm{if}\;m_{x}\neq m_{y}\\ 0, & \;\mathrm{otherwise}\; \end{array}\right. \end{array}$$

where $m_{x}$ and $m_{y}$ represent the virtual machines that process $v_{i}$ and $v_{j}$, respectively. If tasks $v_{i}$ and $v_{j}$ are on the same virtual machine, the data transfer time is 0.

### 3.3. Container Configuration Model

**Figure 2:**
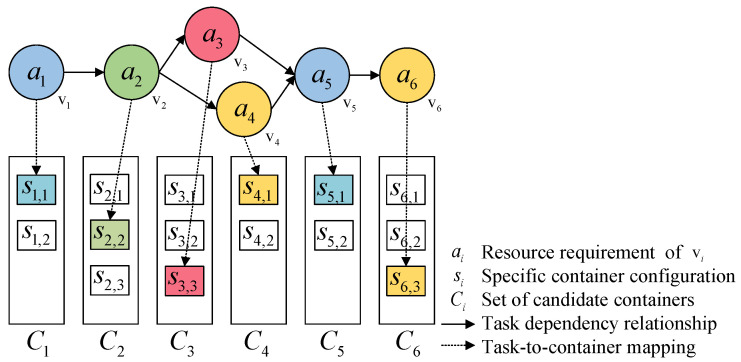
Mapping of resource requirements to container configuration.

Figure 2 is an illustration of container configuration for a microservice workflow. The requirement for task $v_{i}$ in the workflow is represented by $a_{i}$, defined as a tuple $a=(P^{in},P^{out})$. $P^{in}$ represents the required input parameters, and $P^{out}$ represents the output parameters. These requirements are met by specifically configured containers (including hardware parameters, software environment, and dependent data). A container configuration is represented as a tuple $s=\left(ID,P^{in},P^{out},Q\right)$ in the form of container image files. ID is the unique identifier for a specific container image, $P^{out}$ and $P^{in}$ have the same meanings as defined in simple tasks, and *Q* is the Quality of Service (QoS) indicator for the service, represented as a tuple composed of *M* QoS attributes, $Q=\{Q_{1},Q_{2},\ldots ,Q_{M}\}$. This paper considers two QoS attributes: execution time and reliability.

The candidate resource set *C* is a list composed of *K* different resource configuration types, which is represented by $C=\{s_{1},s_{2},\ldots ,s_{K}\}$. These different resources, in the form of container image files, can provide the same functionality with varying service qualities.

The optimization objective is to find resource configuration types that satisfy QoS preferences for a user-submitted workflow request. *w* represents the user’s preferences for QoS attributes, i.e., the weights of different QoS, $w=\{w_{1},w_{2},\ldots ,w_{L}\}$. Among them, $w_{i}$ is greater than 0, and the sum of all $w_{i}$ equals 1.

The QoS values for different service type indicators are weighted and combined using the following formula:

(3) $$\mathrm{QoS}=\sum_{i=1}^{L}w^{i}\times norm\_Q^{i}$$

In this paper, the objective of optimizing reliability is to achieve maximization, whereas the goal for optimizing execution time is to achieve minimization. To unify the measurement of different QoS attributes, *Q* is normalized to norm_Q, calculated using the following formula:

(4) $$norm\_Q^{i}=\left\{\begin{array}{cc} \frac{Q^{i}-min\_Q^{i}}{max\_Q^{i}-min\_Q^{i}}, & \mathrm{if}\;Q^{i}\;\mathrm{is}\;\mathrm{reliability},\\ \frac{max_{\_}Q^{i}-Q^{i}}{max\_Q^{i}-min\_Q^{i}}, & \mathrm{if}\;Q^{i}\;\mathrm{is}\;\mathrm{makespan}. \end{array}\right.$$

where $max\_Q^{i}$ and $min\_Q^{i}$ represent the maximum and minimum values of the *i*-th type of service indicator, respectively.

With QoS as the optimization objective, the problem is defined as follows:

(5) $$\begin{array}{c} \underset{x_{i,j}}{arg max}\;x_{i,j}\times QoS\\ s.t.\;x_{i,j}=0,1,\;i=1,2,\ldots ,N,\;j=1,2,\ldots ,K_{i}\\ \;\sum_{j=1}^{K_{i}}x_{i,j}=1,\;\forall i=1,2,\ldots ,N \end{array}$$

From the mathematical model, it can be seen that the QoS-based container configuration is a classical combinatorial optimization problem.

### 3.4. Failure Model

The time of cloud resource failures follows a Poisson probability distribution [28,29]. Let $\lambda_{l}$ denote the failure rate of virtual machine $m_{l}$ in each time period, which can be obtained by calculating the statistical average based on the historical data of the virtual machine [30]. The reliability of task container $c_{k}$ deployed on virtual machine $m_{l}$ is expressed as:

(6) $$\begin{array}{c} R\left(v_{i},c_{k},m_{l}\right)=e^{-\lambda_{l}\times ET\left(v_{i},c_{k}\right)} \end{array}$$

The fault-tolerant mechanism in our work is based on replication technology, where each task has multiple replicas that can be assigned to containers located on different virtual machines. Let $n_{i}$ denote the number of replicas for task $v_{i}$. The set of replicas for task $v_{i}$ is denoted as $rep\left(v_{i}\right)=\left\{v_{i}^{1},v_{i}^{2},\ldots ,v_{i}^{n_{i}}\right\}$, where $v_{i}^{1}$ is the primary replica and the others are backup replicas. All $n_{i}$ replicas share the same reliability model.

For task $v_{i}$, reliability is calculated as follows:

(7) $$\begin{array}{c} R\left(v_{i}\right)=1-\prod_{\tau =1}^{n_{i}}\left(1-R\left(v_{i}^{\tau },c_{v_{i}^{\tau }},m_{v_{i}}\right)\right) \end{array}$$

For a workflow *W*, reliability is calculated as:

(8) $$\begin{array}{c} R\left(W\right)=\prod_{v_{i}\in V}R\left(v_{i}\right) \end{array}$$

### 3.5. Problem Formulation

The start time of task $v_{i}$ on container $c_{k}$ depends on the availability of container $c_{k}$ and the time when all data from $v_{i}$’s predecessor tasks is received. The formulas for calculating the start time $ST(v_{i},c_{k})$ and finish time $FT(v_{i},c_{k})$ are as follows:

(9) $$\begin{array}{c} ST\left(v_{i},c_{k}\right)=\max\left\{\mathrm{Avail}\left(v_{i},c_{k}\right),\max_{v_{j}\in \mathrm{pred}\left(v_{i}\right)}\right.\\ \left.\left\{\max_{v_{j}^{\tau }\in \mathrm{rep}\left(v_{j}\right)}\left\{FT\left(v_{j}^{\tau }\right)+TT\left(v_{j}^{\tau },v_{i}\right)\right\}\right\}\right\} \end{array}$$

(10) $$\begin{array}{c} FT\left(v_{i},c_{k}\right)=ST\left(v_{i},c_{k}\right)+ET\left(v_{i},c_{k}\right) \end{array}$$

where $rep(v_{j})$ represents the set of replicas of task $v_{j}$.

$\mathrm{Avail}(v_{i},c_{k})$ is the earliest time at which container $c_{k}$ is ready to execute task $v_{i}$ and is calculated using the following formula:

(11) $$\begin{array}{c} \mathrm{Avail}\left(v_{i},c_{k}\right)=\max\left\{IT\left(v_{i},c_{k}\right),\max_{v_{s}\in \mathrm{Sche}\left(c_{k}\right)}\left\{\mathrm{AFT}(v_{s})\right\}\right\} \end{array}$$

where $\mathrm{Sche}(c_{k})$ and $\mathrm{AFT}(v_{s})$ represent the set of tasks assigned to container $c_{k}$ and the actual finish time of task $v_{s}$, respectively.

Additionally, the initialization time $IT(v_{i},c_{k})$ of a task depends on the state of the virtual machine and container and is calculated as follows:

(12) $$\begin{array}{c} IT\left(v_{i},c_{k}\right)=\left\{\begin{array}{cc} 0, & \mathrm{if}\;c_{k}\;\mathrm{is}\;\mathrm{running}\\ IT(c_{k}), & \mathrm{if}\;\mathrm{creating}\;c_{k}\;\mathrm{on}\;m_{l}\\ IT\left(c_{k}\right)+IT\left(m_{l}\right), & \mathrm{if}\;\mathrm{creating}\;c_{k}\;\mathrm{and}\;m_{l} \end{array}\right. \end{array}$$

- If there is a running $c_{k}$ that can handle task $v_{i}$, no initialization is required, i.e., $IT(v_{i},c_{k})=0$.
- If there is no suitable microservice to handle task $v_{i}$, a new container $c_{k}$ is created, and then deploy on an existing virtual machine $m_{l}$. Therefore, initialization of the container is necessary.
- If there is no available microservice to handle task $v_{i}$ and no existing virtual machine, a new container $c_{k}$ and a new virtual machine $m_{l}$ are created.

Based on the above model, the scheduling scheme is represented as π=(V,C,M,ST,ET). $\pi =(v_{i},c_{k},m_{l},ST\left(v_{i},c_{k}\right),FT\left(v_{i},c_{k}\right))$ indicates that task $v_{i}$ is assigned to container $c_{k}$ on virtual machine $m_{l}$ from time $ST(v_{i},c_{k})$ to $FT(v_{i},c_{k})$.

The formulas for calculating $LST(c_{k},m_{l})$ and $LFT(c_{k},m_{l})$ are as follows:

(13) $$\begin{array}{c} \mathrm{LST}\left(c_{k},m_{l}\right)=\min_{v_{i}\in \mathrm{Sche}\left(c_{k}\right)}\left\{ST\left(v_{i},c_{k}\right)\right\} \end{array}$$

(14) $$\begin{array}{c} \mathrm{LFT}\left(c_{k},m_{l}\right)=\max_{v_{i}\in \mathrm{Sche}\left(c_{k}\right)}\left\{FT\left(v_{i},c_{k}\right)\right\} \end{array}$$

The formulas for calculating the total execution time makespan(W) and total execution cost cost(W) of the microservice workflow *W* are:

(15) $$\begin{array}{c} makespan\left(W\right)=\max_{v_{i}\in V}\left\{\max_{v_{i}^{\tau }\in \mathrm{rep}\left(v_{i}\right)}\left\{FT\left(v_{i}^{\tau },c_{v_{i}^{\tau }}\right)\right\}\right\} \end{array}$$

(16) $$\begin{array}{c} cost\left(W\right)=\sum_{m_{l}\in I}p\left(m_{l}\right)\left\lceil \left(\max_{c_{k}\in \mathrm{Sche}\left(m_{l}\right)}\mathrm{LFT}\left(c_{k},m_{l}\right)\right.\right.\left.\left.-\min_{c_{k}\in \mathrm{Sche}\left(m_{l}\right)}\mathrm{LST}\left(c_{k},m_{l}\right)\right)/TU\right\rceil \end{array}$$

where $\mathrm{Sche}\left(m_{l}\right)$ is the set of containers deployed on the virtual machine $m_{l}$, TU represents the minimum unit time for virtual machine leasing, and $p\left(m_{l}\right)$ denotes the BTU cost of the virtual machine $m_{l}$.

Let $D_{req}$ and $R_{req}$ be the deadline and reliability requirements, respectively, for the workflow *W* submitted by the user. The optimization objectives are defined as follows:

(17) $$\begin{array}{c} \min cost(W)\\ s.t.makespan\left(W\right)\leq D_{req}\\ reliability\left(W\right)\geq R_{req}. \end{array}$$

## 4. Proposed Methods

Based on the system architecture and mathematical formulas, this section, respectively, presents methods for the microservice container configuration (DeepMCC) and the reliability microservice workflow scheduling (RMWS).

### 4.1. DeepMCC

**Figure 3:**
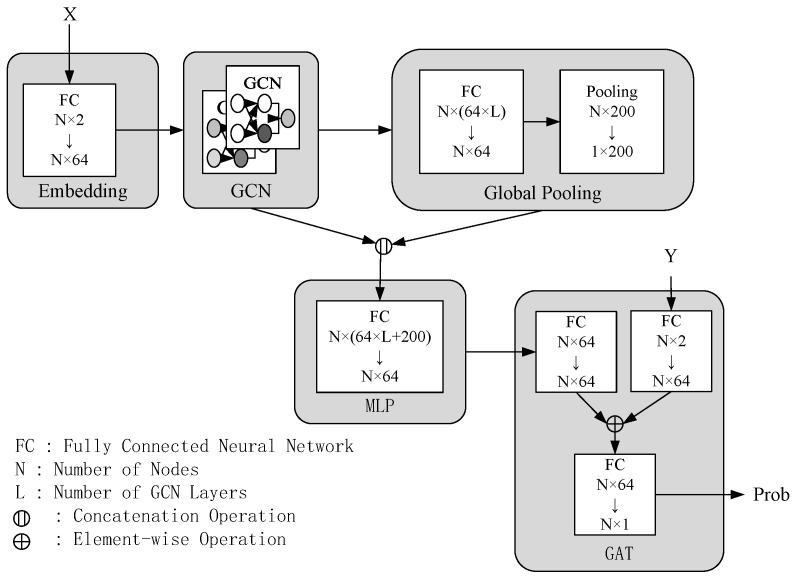
DeepMCC diagram.

The design of DeepMCC is shown in Figure 3, where the model consists of five core blocks: embedding block, GCN block, global pooling block, MLP block, and GAT block. Graph Convolutional Networks (GCN) and Graph Attention Networks (GAT) are integrated into DeepMCC to enhance performance. Specifically, GCN is designed to capture both local and global features of the workflow graph structure. By leveraging multi-layer convolutional operations, it progressively extracts high-level structural features, facilitating a more precise extraction of task relationships and dependencies of the workflow. The GAT block further improves the flexibility and adaptability. By employing an attention mechanism, GAT dynamically assigns weights to nodes or edges based on their importance, enabling the model to handle candidate resource sets of varying sizes. The implementation and training process of DeepMCC are shown in Algorithms 1 and 2.

- Embedding Block: the initial step in task feature processing involves taking the user-submitted workflow graph G=(V,E) as input. Each task node *v* has a candidate configuration set represented as $X_{v}^{qos}$. Edges *E* indicate task dependencies. The embedding block extracts features $x_{v}$ from $X_{v}^{qos}$ through a multilayer perceptron. These feature vectors are transformed by a multi-layer perceptron (MLP) $m_{\theta_{1}}$ to obtain refined feature vectors.
- GCN Block: The feature vectors of each node are updated iteratively. In each iteration, the feature vector of the current node is updated based on the features of its neighbor nodes. After *L* iterations, a set of feature matrices is formed, i.e., $X_{L}=\left\{X_{h}|h=1,2,\ldots ,L\right\}$. These feature matrices are concatenated to form $X_{GCN}$, which contains the updated feature information for all nodes in the graph.
- Global Pooling Block and MLP Block: The Global Pooling Block applies an average pooling operation to $X_{GCN}$ to extract a global feature vector $X_{global}$. This global feature vector is replicated *N* times (where *N* is the number of task nodes) to obtain $X_{repeat}$, which is then concatenated with $X_{GCN}$ to form a feature representation that combines global and local information. The result is fed into an MLP to obtain the final feature representation $X_{GCN^{\prime }}$, integrating both local and global information.
- GAT Block: For each task *v*, the features of its candidate resource set $x_{GCN^{\prime }}^{v}$ and the features of each candidate resource configuration $X_{v,j}^{qos}$ are transformed using multi-layer perceptrons $m_{\theta_{4}}$ and $m_{\theta_{5}}$. An attention coefficient $s_{v,j}$ is calculated using a multi-layer perceptron $m_{\theta_{6}}$ with a Tanh activation function, representing the importance of each candidate resource configuration. The attention coefficients are normalized using the softmax function to obtain the selection probability $Prob_{v,j}$ for each candidate resource configuration.

**Algorithm 1** DeepMCC model.
**Require:** *G* (the workflow graph with requirements), $X_{v}^{qos}$ (the QoS matrix of $s_{v}$ in *G*), *N* (the number of tasks in *G*), *L* (the number of layers in GCN)**Ensure:** 

$Prob_{v,j}$

  1:

$x_{v}\leftarrow \mathrm{ExtractFeatures}\left(X_{v}^{qos}\right)$

  2:

$x_{v}\leftarrow m_{\theta_{1}}\left(x_{v}\right)$

  3:
**for** h=1 **to** *L* **do**
  4:      $x_{v}^{h^{\prime }}\leftarrow \sum_{u\in \mathrm{pred}(v)}m_{\theta_{h^{\prime }}}\left(x_{u}^{h-1}-x_{v}^{h-1}\right)+x_{v}^{h-1}$  5:      $x_{v}^{h}\leftarrow \sum_{u\in \mathrm{succ}(v)}m_{\theta_{h}}\left(x_{u}^{h^{\prime }}-x_{v}^{h^{\prime }}\right)+x_{v}^{h^{\prime }}$  6:      $X_{GCN}\leftarrow X_{GCN}\cup \left\{x_{v}^{h}\right\}$  7:
**end for**
  8:

$x_{\mathrm{global}}\leftarrow \mathrm{POOLING}\left(m_{\theta_{2}}\left(X_{GCN}\right)\right)$

  9:

$X_{\mathrm{repeat}}\leftarrow \mathrm{REPEAT}\left(x_{\mathrm{global}},N\right)$

10:

$X_{GCNConcat}\leftarrow \mathrm{CONCAT}\left(X_{GCN},X_{\mathrm{repeat}}\right)$

11:

$X_{GCN^{\prime }}\leftarrow m_{\theta_{3}}\left(X_{GCNConcat}\right)$

12:

$s_{v,j}^{\prime }\leftarrow m_{\theta_{4}}\left(x_{GCN^{\prime }}^{v}\right)+m_{\theta_{5}}\left(x_{v,j}^{qos}\right)$

13:

$s_{v,j}\leftarrow m_{\theta_{6}}\left(s_{v,j}^{\prime }\right)$

14:

$Prob_{v,j}\leftarrow \mathrm{softmax}\left(s_{v,j}\right)$

15:
**return** $Prob_{v,j}$




The system converts the solution into a multi-hot-type integer vector $y=(y_{1},y_{2},\ldots ,y_{m})$. The length of this vector is equal to the number of resource types in all resource sets pointed to by the current workflow task. Here, $y_{i}=0$ indicates that the resource is not selected by the current workflow; meanwhile, all resource types in the same candidate set can only be selected once. The system uses this multi-hot vector *y* as the label data for the network module. For example, the corresponding label data in Figure 2 are y=(1,0,0,1,0,0,0,1,1,0,1,0,0,0,1).
**Algorithm 2** Training of DeepMCC.**Require:** *G* (The workflow graph with requirements), $X_{v}^{qos}$ (The QoS matrix of $s_{v}$ in *G*), *N*(The number of tasks in *G*), *L*(The number of layers in GCN)**Ensure:** θ (The network parameter)  1:**while** stopping criteria is not satisfied **do**  2:      $Prob_{v}\leftarrow \mathrm{DeepMCC}\left(G,X_{v}^{qos},N,L\right)$  3:      $L\leftarrow -\frac{1}{N}\sum_{v=1}^{N}\sum_{i=1}^{K_{v}}y_{v,i}\log{\mathrm{Loss}}_{j}$  4:      $\theta \leftarrow \theta -\eta \nabla_{\theta }L$  5:**end while**  6:**return **θ

Given a training sample with *m* candidate resources, the output of DeepMCC is a vector $Prob=(p_{s_{1}},p_{s_{2}},\ldots ,p_{s_{m}})$, representing selection probabilities for each resource. Conversely, the initial training labels are a binary vector $y=(y_{1},y_{2},\ldots ,y_{m})$, where $y_{i}$ is either 0 or 1. Predicting the connection between a task and a resource is akin to binary classification. For *m* such connections, there are *m* binary classification problems. Each path prediction is independent. This study employs cross-entropy loss for these binary classifications, computed as follows:

(18) $${\mathrm{Loss}}_{j}=-y_{j}\cdot \log\left(p_{s_{j}}\right)-\left(1-y_{j}\right)\cdot \log\left(1-p_{s_{j}}\right)$$

The loss function obtained after combining multiple QoS attributes is:

(19) $$\;L=-\frac{1}{N}\sum_{v=1}^{N}\sum_{i=1}^{K_{v}}y_{v,i}\log{\mathrm{Loss}}_{j}$$

Subsequently, stochastic gradient descent is employed for training, where each iteration involves using the label data of one resource pattern to update the parameters through gradient descent. The computation process is as follows:

(20) $$\theta \leftarrow \theta -\eta \nabla_{\theta }L$$

where $\nabla_{\theta }L$ represents the gradient of the loss function with respect to the parameters to be optimized, as denoted by θ, and η is the pre-defined learning rate step size.

### 4.2. RWMS

The process of RMWS is shown in Algorithm 3 and the searchResource() is shown in Algorithm 4. The reliability requirements submitted by users serve as the reliability constraints for the workflow. This algorithm assigns a subreliability to each task based on the average reliability lower bound (Line 3). The calculation process is as follows:

(21) $$\begin{array}{c} R\left(v_{i}^{req}\right)=\sqrt[{\left|V\right|}]{R_{\mathrm{req}\;}} \end{array}$$

**Algorithm 3** RWMS.**Require:** Workflow *W*, Deadline $D_{req}$, Reliability $R_{req}$**Ensure:** Scheduling scheme π  1:Initialize priority queue tasklist based on task priorities in *W*  2:Initialize empty lists for containers *C*, VMs *M*, and scheduling scheme π  3:Calculate subreliability for each task: $R\left(v_{i}^{req}\right)=\sqrt[{\left|V\right|}]{R_{req}}$  4:**while **tasklist is not empty **do**  5:      $v_{i,j}\leftarrow tasklist.pop\left(\right)$  6:      ${\vec{R}}^{r}\left(i,j\right)\leftarrow \mathrm{DeepMCC}\left(W,v_{i,j}\right)$  7:      $n_{i,j}\leftarrow \mathrm{Calculate}\;\mathrm{number}\;\mathrm{of}\;\mathrm{replicas}\;\mathrm{for}\;v_{i,j}$  8:      **for** $r\leftarrow 1\;\mathrm{to}\;n_{i,j}$ **do**  9:             $c_{k}\leftarrow \mathrm{Earliest}\;\mathrm{container}\;\mathrm{in}\;C$10:             **if** $c_{k}\;\mathrm{not}\;\mathrm{found}$ **then**11:                $c_{k}\leftarrow$ Create container with $D_{req}\left(v_{i,j}\right)$12:                $C.append(c_{k})$13:             **end if**14:             $ES_{i,j}\leftarrow$$\mathrm{searchResource}(v_{i,j},M,{\vec{R}}^{r}\left(i,j\right))$15:             **if** $ES_{i,j}\;\mathrm{is}\;\mathrm{not}\;\mathrm{empty}$ **then**16:                $m_{l}\leftarrow$ Select a VM according to $VS_{RVD}$,$VS_{KLD}$ or $VS_{LBR}$17:             **else**18:                $m_{l}\leftarrow$ Lease a VM according to $VL_{MIN}$,$VL_{RVD}$ or $VL_{KLD}$19:             **end if**20:             $M.append(m_{l})$21:             $\mathrm{Calculate}\;ST\left(v_{i,j},c_{k}\right)\;\mathrm{and}\;FT\left(v_{i,j},c_{k}\right)$22:             $\pi .append(\left(v_{i,j},c_{k},m_{l},ST,FT\right))$23:    **end for**24:**end while**25:**return **π

For each task, it calculates the resource demand using DeepMCC and determines the number of replicas needed to meet the reliability requirement (Lines 6–7). Then, it iteratively processes tasks from the tasklist, starting with the highest priority (Lines 8–23). It then finds or creates a suitable container and searches for an available resource block on existing or newly requested VMs (14–19). Task start and finish times are calculated and appended to the scheduling scheme (Lines 21–22). The process continues until all tasks are scheduled, ensuring that both reliability and resource constraints are respected.
**Algorithm 4** $\mathrm{searchResource}(v_{i,j},M,{\vec{R}}^{r}\left(i,j\right))$.  1:Initialize $ES_{i,j}\leftarrow ⌀$  2:**for **$m_{k}\in M$** do**  3:      $t_{1}\leftarrow \mathrm{current}\;\mathrm{time}\;\mathrm{or}\;est_{j}^{i}$  4:      **while** $t_{1}\leq D_{req}\left(v_{i,j}\right)-T_{i,j}^{e}$ **do**  5:            flag←true, $t_{2}\leftarrow t_{1}$  6:            **while** $t_{2}\leq t_{1}+T_{i,j}^{e}$ **do**  7:                  **if** ${\vec{R}}^{f}\left(m_{k},t_{2}\right)\geq {\vec{R}}^{r}\left(i,j\right)$ **then**  8:                         $t_{2}\leftarrow t_{2}+1$  9:                  **else**10:                         flag←false11:                         $t_{1}\leftarrow t_{2}$12:                         **break**13:                  **end if**14:            **end while**15:            **if** flag **then**16:                  Add $\left(m_{k},t_{1}\right)$ to $ES_{i,j}$17:            **end if**18:            $t_{1}\leftarrow t_{1}+1$19:      **end while**20:**end for**21:**return **$ES_{i,j}$

#### 4.2.1. Heuristic Strategies

Three heuristic strategies are designed for container placement and virtual machine leasing, respectively. When $ES_{i,j}$ is non-empty, it signifies the need to search for an available virtual machine (VM) within the leased instances to deploy the container for task $v_{i,j}$, while adhering to time constraints and reliability requirements. The feasible start time for task $v_{i,j}$ is within $\left[est_{j}^{i},d\left(i,j\right)-T_{i,j}^{e}\right]$, and the chosen VM’s idle resources must suffice for $v_{i,j}$’s demands during $\left[T_{i,j}^{s},T_{i,j}^{f}\right)$. The suitable VM is selected based on resource similarity and load balancing, considering the resource request vector ${\vec{R}}^{r}\left(i,j\right)$ and VM’s remaining resources ${\vec{R}}^{f}\left(k,t\right)$. Three formulas are used to calculate the distance between resource vectors:

- Resource Vector Distance (RVD):
  (22) $$\begin{array}{c} RVD\left({\vec{R}}^{f}\left(k,t\right),{\vec{R}}^{r}\left(i,j\right)\right)=\sum_{l=1}^{m}{\left(x_{l}\left(k,t\right)-y_{l}\left(i,j\right)\right)}^{2} \end{array}$$
  where $x_{l}\left(k,t\right)$ and $y_{l}\left(i,j\right)$ represent the proportions of resource types in ${\vec{R}}^{f}\left(k,t\right)$ and ${\vec{R}}^{r}\left(i,j\right)$, respectively.
- Kullback–Leibler Distance (KLD):
  (23) $$\begin{array}{c} KLD\left({\vec{R}}^{f}\left(k,t\right),{\vec{R}}^{r}\left(i,j\right)\right)=\sum_{l=1}^{m}x_{l}\left(k,t\right)\cdot \ln\frac{x_{l}\left(k,t\right)}{y_{l}\left(i,j\right)} \end{array}$$
- Load Balancing Rate (LBR):
  (24) $$\begin{array}{c} LBR\left({\vec{R}}_{l}^{f}\left(k,t\right),{\vec{R}}_{l}^{r}\left(i,j\right)\right)=\sum_{l=1}^{m}\sqrt{{\left(ratio\left({\vec{R}}_{l}^{a}\left(k\right)\right)-ratio\left({\vec{R}}_{l}^{a}\left(k\right)\right)\right)}^{2}} \end{array}$$
  where $ratio({\vec{R}}_{l}^{a}\left(k\right))$ and $ratio({\vec{R}}_{l}^{f}\left(k\right),{\vec{R}}_{l}^{r}\left(i,j\right))$ denote the resource proportions.

Based on the three criteria of distance, similarity, and load balancing, the following heuristic virtual machine selection rules are designed:

- $VS_{RVD}$: Select the VM in $ES_{i,j}$ with the smallest RVD;
- $VS_{KLD}$: Select the VM in $ES_{i,j}$ with the smallest KLD;
- $VS_{LBR}$: Select the VM in $ES_{i,j}$ with the smallest LBR.

Three heuristic rules for leasing virtual machines are designed when $ES_{i,j}$ is Empty:

- $VL_{MIN}$: Lease the VM for container $c_{k}$ with the lowest cost;
- $VL_{RVD}$: Lease the VM for container $c_{k}$ with the smallest RVD;
- $VL_{KLD}$: Lease the VM for container $c_{k}$ with the smallest KLD.

#### 4.2.2. Improving the Scheduling Solution

**Figure 4:**
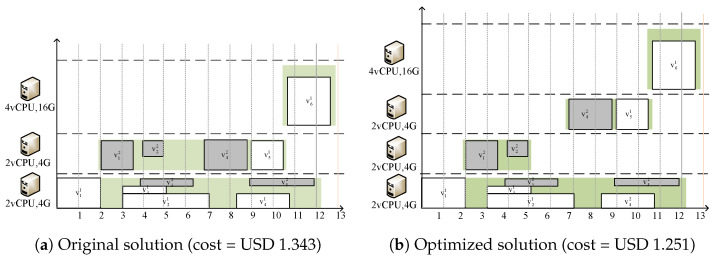
Instance of scheduleImprove.

After generating the initial task scheduling solution π, Algorithm 5 is designed to optimize cost. The algorithm reduces virtual machine fragmentation through container migration. Algorithm 6 sequentially searches for idle slots on virtual machines. If a slot longer than the startup time is found, it will lease a new virtual machine and migrate the eligible container to the new virtual machine. Figure 4 illustrates a scheduling instance as an example.
**Algorithm 5 **scheduleImprove.  1:Initialize M as the set of all leased VMs.  2:**for** each $m_{k}\in M$ **do**  3:      ${\mathrm{TS}}_{k}\leftarrow$ Call idleTimeSlots**()** for $M_{k}$  4:      **for** each time slot $\mathrm{TS}\in {\mathrm{TS}}_{k}$ **do**  5:            $t_{s}\leftarrow$ start time of slot TS  6:            $t_{e}\leftarrow$ end time of slot TS  7:            **if** $t_{e}-t_{s}>T_{k}^{s}$ **then**  8:                  Lease a new VM instance, $M_{new}$  9:                  Migrate all containers scheduled in $M_{k}$ when $T_{i,j}^{s}>t_{e}$ to $M_{new}$.10:                  Update lease time and release time of $M_{new}$11:                  Add $M_{new}$ to M12:            **end if**13:      **end for**14:**end for**15:Update π16:**return **π
**Algorithm 6 **idleTimeSlots.  1:**for **i=1** to ***n*** do**  2:      **for** j=1** to **$\mu_{i}$ **do**  3:            Add task $v_{i,j}$ to list L  4:      **end for**  5:**end for**  6:Sort L by task start times $T_{i,j}^{s}$ in ascending order.  7:Initialize an empty set for time slots, ${\mathrm{TS}}_{k}\leftarrow ⌀$  8:Set initial variables: v←L[0], $t_{s}\leftarrow T^{f}\left(v\right)$, and $t_{e}\leftarrow T^{f}\left(v\right)$  9:Define $t_{max}$ as the maximum task finish time in the task list10:**while **$t_{s}\leq t_{max}$ and $t_{e}\leq t_{max}$ **do**11:      **if** $\sum_{v_{i,j}\in L}\left\{y_{i,j,k}\left(t_{s}\right)\right\}>0$ **then**12:            $t_{s}\leftarrow$ select maximum $T_{i,j}^{f}$ in which $y_{i,j,k}\left(t_{s}\right)=1$13:      **else**14:            $t_{e}\leftarrow t_{s}+1$15:            **while** $\sum_{v_{i,j}\in L}\left\{y_{i,j,k}\left(t_{e}\right)\right\}=0$ and $t_{e}\leq t_{max}$ **do**16:                  $t_{e}\leftarrow t_{e}+1$17:            **end while**18:            **if** $t_{e}>t_{s}$ **then**19:                  Add the time slot $\left(t_{s},t_{e}\right)$ to ${\mathrm{TS}}_{k}$20:                  $t_{s}\leftarrow t_{e}$21:            **end if**22:      **end if**23:**end while**24:**return** the set of identified time slots, ${\mathrm{TS}}_{k}$

## 5. Experiment

### 5.1. Experimental Setting

Due to the scarcity of open-source datasets specifically tailored for microservice scenarios, this paper utilizes simulated data to conduct its experiments. The following details the data processing and generation methods employed:

- Container Configuration Data: The foundation of our container configuration data are the Quality of Web Service dataset [31], which comprises 2507 real-world web service entries. To create a structured resource set, we applied text clustering techniques to these entries, resulting in 200 distinct clusters. Each cluster serves as a candidate resource set, with sizes varying from as few as 2 to as many as 200 entries, providing a diverse range of options for our simulations.
- Microservice Application Datasets: Our microservice application datasets are derived from four reputable scientific workflow datasets: Cybershake, LIGO, Montage, and SIPHT. These datasets encompass workflows with varying complexities, with the number of tasks in a single workflow ($\mu_{i}$) ranging from 100 to 1000 in increments of 100. This wide range allows us to test the robustness and scalability of our proposed methods across different workload sizes.
- Training Samples for DeepMCC: We configured the generator to produce workflows with task counts ranging from 20 to 100, ensuring a diverse set of training instances. A Genetic Algorithm (GA) was then employed to determine near-optimal solutions for these workflows, which served as our training and validation data samples. For each sample size within the specified range, we generated 1000 training samples, 100 validation samples, and 50 test samples randomly.
- User-Defined Deadlines and Reliability Requirements: To simulate real-world user constraints, we set user-defined deadlines ($D_{i}$) based on the earliest finish time ($eft_{\mu_{i}}^{i}$) of each workflow. These deadlines were calculated by multiplying $eft_{\mu_{i}}^{i}$ by a factor θ, which ranged from 0.02 to 0.2 in increments of 0.02. Similarly, user-defined reliability requirements ($R_{req}$) were varied from 0.7 to 0.999 in increments of 0.05, allowing us to assess the model’s performance under different reliability constraints.
- Virtual Machine Prices: The prices of virtual machines used in our simulations are based on the billing model of Amazon Elastic Container Service (Amazon ECS) [32]. A detailed table (Table 3) outlines the unit costs of various on-demand AWS EC2 instances, providing a realistic pricing structure for our cost-optimization analyses [32].

### 5.2. Performance Evaluation of DeepMCC

To verify the effectiveness, DeepMCC makes a comparison with three combinatorial optimization algorithms: the Multiple Population Genetic Algorithm (MPGA) [33] as a representative of metaheuristic approaches, the Double Deep Q-Network algorithm (DDQN) [34] as a deep reinforcement learning approach, and a pre-trained deep reinforcement learning algorithm with QoS-labeled data (QoS-DRL). The hyperparameters for each algorithm are summarized in Table 4.

**Figure 5:**
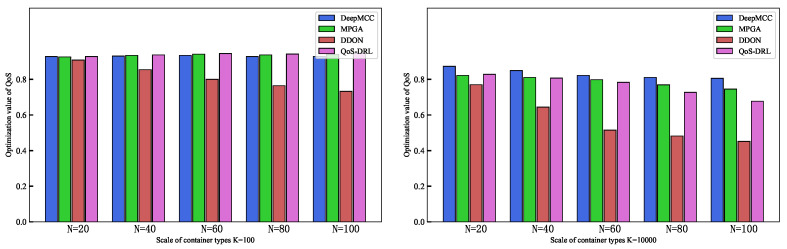
Average QoS value for different workflow sizes.

Each algorithm is run 10 times on the test samples, and the average QoS value is the solution result. The experimental results for different *K* values are shown in Figure 5. Table 5 and Table 6 present the average QoS and runtime for the algorithms under different workflow sizes (*N*) and resource sizes (*K*), respectively.

The QoS value of all algorithms gradually decrease with an increasing number of workflow nodes. Specifically, when *K* is small, DeepMCC, MPGA, and QoS-DRL achieve higher QoS values. However, as the number of workflow nodes increases, DDQN lags behind DeepMCC, MPGA, and QoS-DRL. When K=10,000, DeepMCC and MPGA perform the best. QoS-DRL decrease when the number of workflow nodes is large, but it still outperforms DDQN.

**Figure 6:**
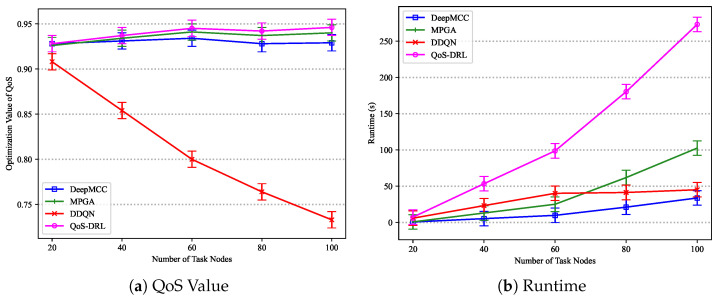
The trends of QoS value and Runtime with varying number of nodes among different algorithms (*K* = 100).

Figure 6 shows the trends in QoS optimization and runtime for K=100. When the number of the candidate resource is small (K=100), DeepMCC exhibits a relatively short runtime with an increasing number of tasks. In contrast, MPGA has a significantly longer runtime compared to DeepMCC, making it the slowest among the four algorithms. DDQN shows a longer runtime for smaller task sizes but becomes relatively faster as the task size increases. QoS-DRL consistently demonstrates the longest average runtime. As the task size grows, MPGA performs the best.

**Figure 7:**
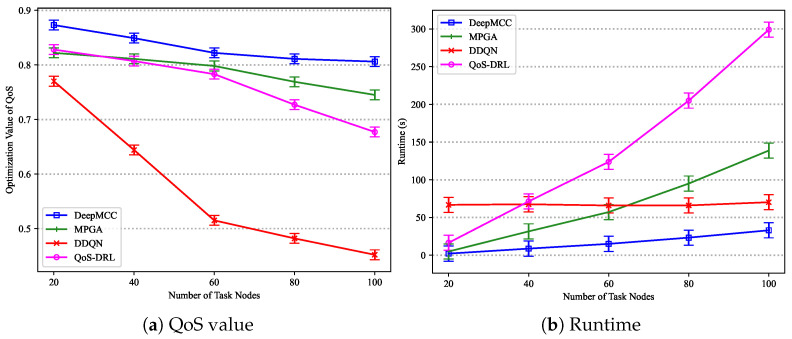
The trends of QoS value and runtime with varying number of nodes among different algorithms (K = 10,000).

Figure 7 shows the trends for K=10,000. As the candidate resource increases (K=10,000), the performance of the algorithms varies significantly. DeepMCC remains the most advantageous algorithm, with a relatively slow increase in runtime as the workflow size grows. MPGA has a significantly longer runtime compared to DeepMCC. DDQN’s runtime varies little across different workflow sizes but is generally longer. QoS-DRL has the longest average runtime.

Summarizing the experimental results, DeepMCC outperforms MPGA by an average of 2.18% in QoS optimization, surpasses DDQN by 27.2%, and surpasses upon QoS-DRL by 3.37%. In terms of execution time, DeepMCC reduces runtime by an average of 71.2% compared to MPGA, 69.9% compared to DDQN, and 88.49% compared to QoS-DRL.

### 5.3. Performance Evaluation of RMWS

Three algorithms were used as baselines for comparison with RMWS: ProLiS [3], IRW [35], and CCRH [36]. ProLiS introduces a probabilistic task sorting and subdeadline allocation method to enhance fairness and efficiency in task scheduling. However, ProLiS does not consider reliability requirements. Therefore, we extend the reliability method to ProLiS by iteratively assigning task replicas to virtual machines until the subdeadlines are met. Two representative fault-tolerant scheduling algorithms, IRW and CCRH, are selected to evaluate the fault-tolerance performance. Both algorithms consider fault tolerance in scheduling, with IRW based on a resubmission strategy and CCRH employing a replication strategy. However, neither IRW nor CCRH considers deadline constraints. Therefore, the task sorting processes of IRW and CCRH are modified to prioritize tasks according to the subdeadline partitioning strategy.

**Figure 8:**
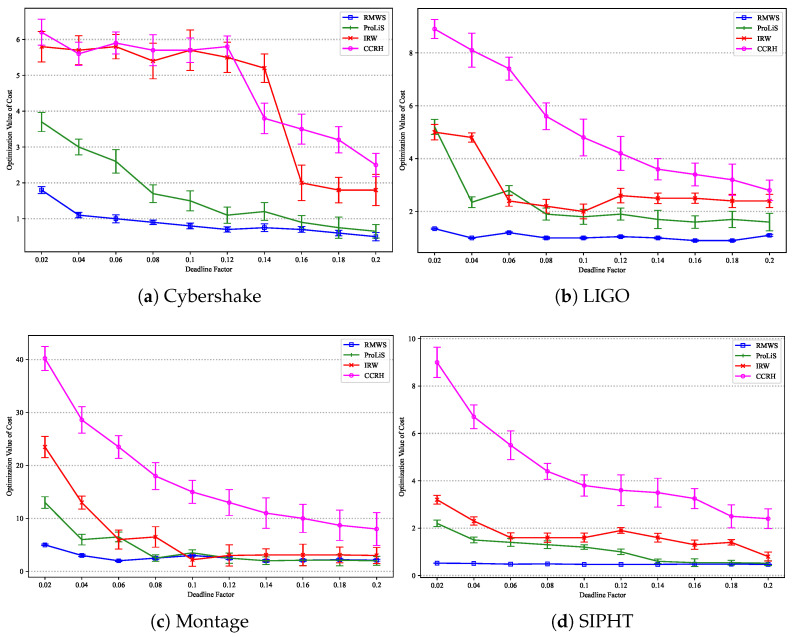
Cost optimization results under different deadlines.

Figure 8 illustrates the trends in execution cost as the deadline factor θ varies for the four algorithms on the Cybershake, LIGO, Montage, and SIPHT datasets, respectively. It is observed that the execution cost decreases as θ increases, allowing algorithms to utilize cheaper virtual machine resources to reduce costs.

When θ is greater than 0.16, RMWS and ProLiS exhibit similar optimization performance. However, as θ increases beyond 0.16, ProLiS’s cost optimization ability gradually diminishes, widening the gap with RMWS, though it still outperforms IRW and CCRH. RMWS consistently demonstrates the best and most stable cost optimization on the Cybershake dataset, meeting cost optimization targets under various deadline constraints. Notably, cost reduction becomes ineffective for IRW and CCRH when θ falls below a certain threshold. Specifically, IRW execution cost surges when θ is less than 0.15, remaining high thereafter. Similarly, CCRH exhibits the same phenomenon when θ is less than 0.13. The significant deviation in trends between IRW, CCRH, and the other three workflow datasets on Cybershake may be attributed to Cybershake’s abundant parallel structures, which require more virtual machines to accommodate tasks, increasing the number of virtual machines and leading to more resource contention and fragmentation.

Due to the cost surge of IRW and CCRH on the Cybershake dataset under tight deadline constraints (θ<0.13), their data are excluded from this experiment’s statistics. Across the three workflow application types (LIGO, Montage, and SIPHT), RMWS reduces execution costs by an average of 32.70%, 49.50%, and 73.72% compared to ProLiS, IRW, and CCRH, respectively.

**Figure 9:**
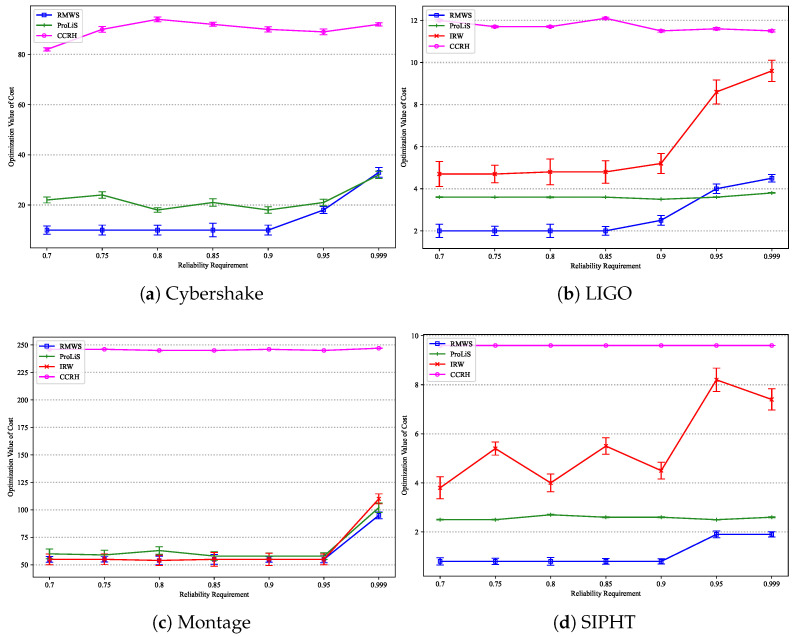
Cost optimization results under different reliability constraints.

Figure 9 presents the execution costs under various reliability constraints for the four algorithms on the Cybershake, LIGO, Montage, and SIPHT datasets, respectively. On the Cybershake dataset, IRW consistently fails to meet reliability requirements, so only ProLiS, CCRH, and RMWS are analyzed. CCRH incurs the highest cost, while the execution costs of the other algorithms increase slightly with $R_{req}$. When $R_{req}$ exceeds 0.99, the execution costs of all algorithms surge dramatically. This is because when $R_{req}$ is low, reliability demands are easily met, with the primary constraint being the deadline. However, when $R_{req}$ exceeds a certain threshold, reliability demands become the primary obstacle, necessitating the leasing of additional cloud resources to deploy more task replicas. Since CCRH’s strategy selects the maximum number of replicas per task for reliability, its execution cost is the highest.

Although IRW fails to find feasible solutions meeting constraints on the Cybershake dataset, it outperforms ProLiS on the other three datasets. The performance of IRW under reliability requirements is most closely related to workflow type, with the most significant impact from workflow structure variations observed on the SIPHT dataset. Across all workflow applications, RMWS improves execution costs by 26.63%, 35.67%, and 70.03% compared to ProLiS, IRW, and CCRH, respectively.

**Figure 10:**
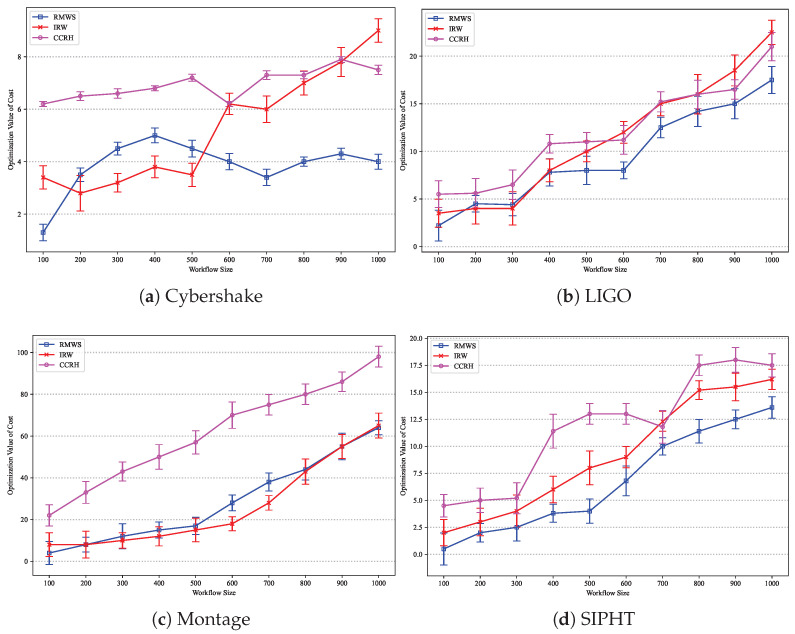
Cost optimization result under different workflow sizes.

Figure 10 shows the execution cost optimization results for different workflow sizes on the Cybershake, LIGO, Montage, and SIPHT datasets, respectively. As workflow size increases, the execution costs of all algorithms rise. When the workflow size range from 200 to 500, IRW outperforms other algorithms. However, for larger workflows (e.g., greater than 500), IRW’s performance on the SIPHT dataset is inferior to RMWS. RMWS demonstrates the best cost optimization ability in larger workflow sizes. Compared to IRW and CCRH, RMWS reduces the average cost by 27.84% and 47.57%, respectively.

## 6. Conclusions and Future Work

This paper considers the cost optimization of microservices workflows with deadlines and reliability on container environment. DeepMCC is designed to learn from resource combinations to predict container selection probabilities. Additionally, a reliability scheduling algorithm RMWS is proposed with replication-based task scheduling and container migration process, which reduces VM leasing costs by utilizing idle fragments. Experimental results demonstrate that the DeepMCC supports solve the configuration problem of large-scale tasks effectively, and the proposed RWMS exhibits high performance compared to the comparison algorithms. Specifically, the DeepMCC model reduces the average runtime by 76.53% compared to three comparison algorithms, and the RWMS achieves an average improvement of 44.59% in cost optimization compared to the three contrast algorithms. Future work includes validating the proposed algorithms in real-world data, and utilizing unsupervised learning models to overcome the limitations of pre-training.

## Figures and Tables

**Table 1:** Related literature on microservice workflow scheduling.

Literature	Makespan/Deadline	Cost/Budget	Reliability	Algorithm
Bao et al. [13]	✓	✓		Heuristic
Wang et al. [14]	✓	✓		Heuristic
Li et al. [15]	✓	✓	✓	Heuristic
Abdullah et al. [16]		✓		Deep Learning
Yu et al. [17]			✓	Reinforcement Learning
Our work	✓	✓	✓	Graph Deep Learning & Heuristic

**Table 2:** Description of main symbols.

Symbol	Description
*W*	Microservice workflow
*V*	Set of tasks in *W*
*E*	Set of task dependencies in *W*
$v_{i}$	The *i*-th task in *W*
$w_{i}$	Computational workload of $v_{i}$
$e_{i,j}$	Edge between $v_{i}$ and $v_{j}$
*M*	Set of virtual machines
$m_{l}$	The *l*-th virtual machine in *M*
$p(m_{l})$	Price of virtual machine $m_{l}$
*C*	set of candidate containers
$c_{k}$	The *k*-th container in *C*
$\vec{R}\left(c_{k}\right)$	Resource vector of $c_{k}$
${\vec{R}}^{a}\left(k\right)$	Resource vector of $m_{k}$
$ET(v_{i},c_{k})$	Execution time of $v_{i}$ on $c_{k}$
$ST(v_{i},c_{k})$	Start time of task $v_{i}$ on $c_{k}$
$FT(v_{i},c_{k})$	Finish time of task $v_{i}$ on $c_{k}$
$a_{i}$	Requirement parameters of $v_{i}$
$s_{i,j}$	The *j*-th candidate container of $v_{i}$
*Q*	Quality of Service (QoS)
*w*	Weights of user’s QoS preferences
$x_{i,j}$	decision variable for task-to-container
$Prob_{i,j}$	probability of task-to-container
$R(v_{i},c_{k},m_{l})$	Reliability of $v_{i}$ in $c_{k}$ on $m_{l}$
$rep(v_{i})$	Set of replicas for $v_{i}$
π	scheduling scheme

**Table 3:** Amazon EC2 pricing.

Instance Type	CPU Cores	Memory	BTU
m5.4xlarge	16vCPU	64 GB	USD 0.7680
m5.2xlarge	8vCPU	32 GB	USD 0.3840
m4.xlarge	4vCPU	16 GB	USD 0.2000
m4.large	2vCPU	8 GB	USD 0.1000
t2.medium	2vCPU	4 GB	USD 0.0464
t2.small	1vCPU	2 GB	USD 0.0230

**Table 4:** Hyperparameter settings for the algorithms.

Algorithm	Hyperparameters
DeepMCC	batch_size: 16, learning_rate: 0.0005, epoch_num: 30, gnn_layer: 6
MPGA	population_size: 100, group_num: 4, cross_rate: 0.5, epoch: 600
DDQN	frames: 10,000, batch_size: 32, buffer_size: 10,000, learning_rate: 0.01, GAMMA: 0.9, min_eps: 0.01, max_eps: 0.9, eps_frames: 10,000, sampling_weight: 0.4, Q_updates_num: 500
QoS-DRL	iter_num: 10, pretrain_epoch: 360, learning_epoch: 300, drl_lr: 0.0001, pretrain_lr: 0.001, sample_num: 64, best_num: 64

**Table 5:** Average QoS value.

Algorithm	*K*	*N*				
		**20**	**40**	**60**	**80**	**100**
DeepMCC	100	0.928	0.931	0.934	0.928	0.929
	1000	0.9	0.891	0.879	0.871	0.868
	10,000	0.873	0.849	0.822	0.811	0.806
MPGA	100	0.926	0.934	0.941	0.937	0.94
	1000	0.874	0.873	0.87	0.854	0.843
	10,000	0.822	0.811	0.798	0.769	0.745
DDQN	100	0.908	0.854	0.8	0.764	0.733
	1000	0.839	0.75	0.658	0.623	0.593
	10,000	0.77	0.644	0.515	0.482	0.452
QoS-DRL	100	0.928	0.937	0.945	0.942	0.946
	1000	0.878	0.873	0.865	0.836	0.812
	10,000	0.828	0.807	0.783	0.727	0.677

**Table 6:** Average runtime (unit: seconds).

Algorithm	*K*	*N*				
		**20**	**40**	**60**	**80**	**100**
DeepMCC	100	0.653	5.259	9.785	21.112	33.75
	1000	1.417	7.023	12.455	21.907	33.177
	10,000	2.154	8.757	15.053	23.318	33.042
MPGA	100	0.756	13.023	25.09	61.922	102.598
	1000	2.877	22.347	41.261	77.37	119.899
	10,000	4.945	31.626	57.198	95.027	138.799
DDQN	100	5.88	23.204	40.173	41.345	45.084
	1000	36.676	45.509	53.212	53.054	57.298
	10,000	66.786	67.55	65.946	66.047	70.28
QoS-DRL	100	7.298	53.418	98.72	180.362	273.203
	1000	12.006	62.568	111.575	190.02	284.231
	10,000	16.491	71.387	123.782	204.99	299.02

## Data Availability

The datasets used in this study are available from the corresponding author upon reasonable request. These data were used under license for the current study and cannot be redistributed without permission from the data provider.
